# Knowledge of lateralized brain function can contribute to animal welfare

**DOI:** 10.3389/fvets.2023.1242906

**Published:** 2023-08-04

**Authors:** Lesley J. Rogers

**Affiliations:** School of Science and Technology, University of New England, Armidale, NSW, Australia

**Keywords:** hemispheric asymmetry, limb preference, cognitive bias, fear, stress, farm animals, companion animals, welfare

## Abstract

The specialized functions of each hemisphere of the vertebrate brain are summarized together with the current evidence of lateralized behavior in farm and companion animals, as shown by the eye or ear used to attend and respond to stimuli. Forelimb preference is another manifestation of hemispheric lateralization, as shown by differences in behavior between left- and right-handed primates, left- and right-pawed dogs and cats, and left- and right-limb-preferring horses. Left-limb preference reflects right hemisphere use and is associated with negative cognitive bias. Positive cognitive bias is associated with right-limb and left-hemisphere preferences. The strength of lateralization is also associated with behavior. Animals with weak lateralization of the brain are unable to attend to more than one task at a time, and they are more easily stressed than animals with strong lateralization. This difference is also found in domesticated species with strong vs. weak limb preferences. Individuals with left-limb or ambilateral preference have a bias to express functions of the right hemisphere, heightened fear and aggression, and greater susceptibility to stress. Recognition of lateralized behavior can lead to improved welfare by detecting those animals most likely to suffer fear and distress and by indicating housing conditions and handling procedures that cause stress.

## 1. Introduction

Brain function in vertebrate species is lateralized, meaning that the left and right sides of the brain process sensory inputs in different ways and control different types of behavior (1, 2). In species with eyes in a lateral position, inputs from each eye cross the midline and reach the opposite side of the brain. Although, in birds at least, there is some minor recrossing of the midline of the brain as visual information is fed forward from the retinal recipient region to the forebrain hemispheres, by far the main inputs are fed forward to the forebrain without crossing the midline and processing is carried out by the hemisphere opposite the “seeing eye” (3, 4). Consequently, lateralization of visual function can be revealed by testing animals monocularly either by applying a patch to one eye and then the other eye or by presenting a stimulus in the lateral, monocular visual field of one eye or the other eye (5).

**Figure 1:**
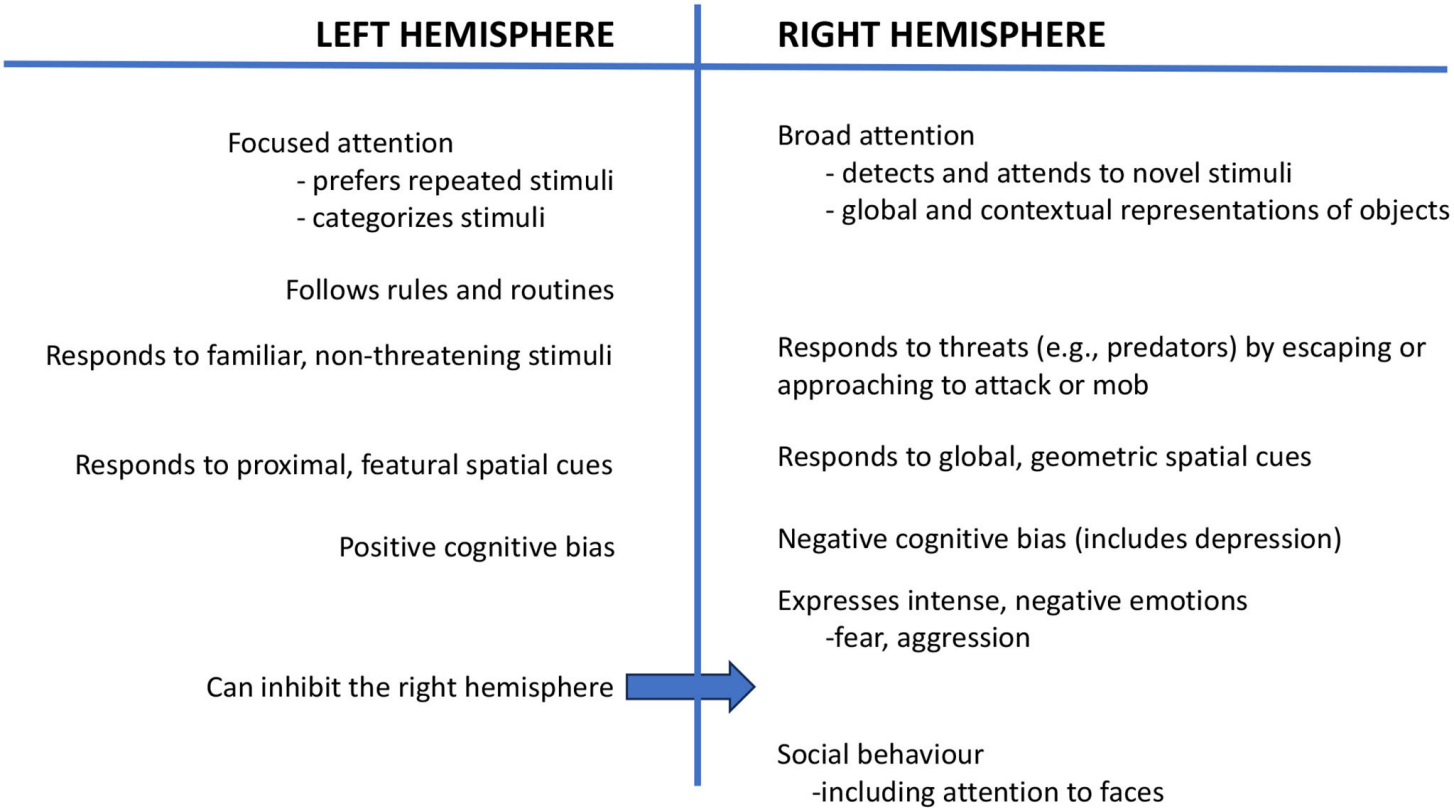
This figure presents a summary of the functions of the left and right hemispheres, as reported in a range of species.

Using eye occlusion and testing young chicks, a body of research has shown that the left hemisphere, which receives input from the right eye, categorizes stimuli, as needed to distinguish food grains from a background of pebbles (6, 7), and consistent with this, the left hemisphere focuses attention and attends to repeated and familiar stimuli (1) (Figure 1). In contrast, the right hemisphere (left eye) has broad attention and detects novel stimuli (8, 9), including predators (10), and it controls the expression of strong emotions, including fear and aggression (11). The right hemisphere is also used in social behavior (12) and sexual behavior (11) (Figure 1). Both of the hemispheres are also specialized to attend to different aspects of spatial information: The left hemisphere attends to the proximal, landmark features of stimuli, whereas the right hemisphere deals with global spatial information and geometric cues (13).

Mounting evidence shows that these specializations of hemispheric function are also characteristic of farm animals (14, 15). Sheep, for example, process faces using their right hemisphere, clearly an aspect of social behavior (16, 17). Horses show higher levels of aggression to conspecifics that they see with their left eye, and thus, when they are using their right hemisphere (18). In addition, as electroencephalogram measurements have shown, horses use their right hemisphere when attending to a novel stimulus (19), and they have a strong preference to view novel objects with the left eye (20). Cows that use their left eye and right hemisphere are more anxious/fearful (21, 22), and consistent with this, submissive cows use their left eye when viewing dominant cows (23). Generally, in vertebrate species, including horses and sheep, infants position themselves on their mother's right side and thereby monitor her behavior with their left eye and right hemisphere (24, 25). In adult, feral horses grazing in pairs with one leading and another following, there is a side bias for the leading horse to use its left eye and right hemisphere to monitor the follower (26). Similarly, elands position themselves so that the nearest herd mates are on their left side and thus can be monitored using the right hemisphere (27). These interactions using the right hemisphere are consistent with the specialization of the right hemisphere for social behavior (28, 29), which can involve dominance and aggression (18) or an affiliative approach (30).

Similar right hemispheric specializations have been found in companion animals responding to visual and auditory stimuli. Dogs that were presented with visual images in the left monocular, visual field respond more strongly to a fear-inducing stimulus than they do when the same stimulus is presented in the right visual field, which means that the right hemisphere is used (31). The right hemisphere is also used by dogs to process human vocalizations with negative emotional valence (31, 32) and the threatening sounds of a thunderstorm (33). Cats too use the right hemisphere to respond to vocalizations eliciting fear, as happens when they hear a barking dog (34). As shown in cats, the stress response, measured as elevated levels of cortisol, occurs together with elevated temperature of the right ear only (35). This demonstrates specialized activation of the right hemisphere during stress behavior since elevated neural activity increases blood flow to that hemisphere and that causes an increase in the temperature of the ear on the same side.

## 2. Discussion

A general pattern of vertebrates to respond negatively to stimuli on their left side suggests potential ways of improving the welfare of species used in agriculture (14). For example, in practices that require farm animals to move along corridors or runways, it would be desirable to ensure that no unfamiliar or fear-inducing stimuli are present on the left side. Considering laterality might also improve the handling of animals. For example, horses may be more fearful of unfamiliar handlers approaching on their left side. Some empirical data support this suggestion: Horses were found to be more fearful of a person opening an umbrella while approaching on their left side than when this approach was on their right side (36), and untrained young horses expressed more negative behavior (escaping and threatening) when approached on their left side (37).

### 2.1. Limb preference and cognitive bias

As an extension of the above, some types of motor performance can indicate which hemisphere an animal is using. For example, an animal with a preference to use its right forelimb may respond differently from one with a preference to use its left limb because the former has a bias to use its left hemisphere, and the latter has a bias to use its right hemisphere. Consequently, one can predict that animals with a left-limb preference would display negative responses to novel stimuli, whereas those with a right-limb preference would display positive responses to the same stimuli. In humans, these behavioral responses are referred to as pessimism vs. optimism and, hence, as cognitive judgment bias, or simply as cognitive bias. Tests of judgment or cognitive bias have been developed for non-human species, thus providing information about the cognitive state of animals as a measure of their welfare (38).

As an example of limb/hand preference reflecting cognitive bias, marmosets with left-hand vs. right-hand preferences to pick up and hold food were tested on a cognitive bias task (39). First, they were trained to expect to find a food reward inside a bowl covered with a white lid and not in a bowl with a black lid (or vice versa). They were then tested by presenting a bowl with an ambiguous, gray lid. Right-handed marmosets responded to the ambiguous bowl as if it was positive (i.e., contained food) by approaching it and removing the lid to look inside the bowl, whereas left-handed marmosets treated it as negative (i.e., did not approach). Right-handed marmosets have also been found to touch more objects located in unfamiliar surroundings than do left-handed marmosets (40). Closely similar results have been obtained by testing Geoffroy's marmosets with a range of stimuli: The left-handed ones were more fearful of a predator's vocalizations and were more reluctant to sample novel foods (41). A recent study of a group of mixed species of primates, including marmosets and tamarins, found similarly that left-handed subjects inspected fewer novel objects than right-handed ones (42). This result is consistent with fear being higher in left-handed primates. In addition, a recent study by Barbary macaques has reported that a left-hand preference is associated with higher levels of fear and tension in response to predators (43). Left-handed marmosets also express fewer social interactions with conspecifics than do right-handed marmosets (44, 45).

These results show that left-hand preference is associated with negative cognitive bias. Future research should investigate whether left-hand or left-limb preference is associated with a propensity to display a behavior indicative of depression, as has been shown in rats (46), and also with increased behavioral despair, shown in rats as immobility in the forced swim test (47). Clearly, these results have implications for animal welfare.

In primates, hand preference can be used to assess the likelihood of an individual being stressed, or distressed, by housing conditions or conspecifics. Limb preference in species used in agriculture may also indicate susceptibility to social stress or negative response to conditions of confinement and handling. For example, horses show a relationship between limb preference and cognitive bias. Marr et al. (48) found that those with a preference to commence moving off with the left forelimb (the right hemisphere in control) are more likely to respond to an ambiguous stimulus (one placed midway between a location trained to be rewarding and a location not rewarded) as negative than are horses with a right-forelimb preference. A similar association between paw preference and cognitive bias has been found in dogs. Wells et al. (49) scored paw preference as the preferred paw to hold down a Kong baited with food, and then, they trained the dogs to expect a food reward in a bowl placed at one location. They were then tested with a bowl placed at a short distance from the previously rewarded location. Left-pawed dogs were more reluctant to approach the bowl in the new location than were right-pawed dogs or ambilateral dogs (49). This finding suggests that negative cognitive bias depends on the exclusive use of the right hemisphere and that the use of the left hemisphere suppresses this negativity. In addition, the balanced use of the hemispheres, as in ambilateral dogs, seems to suppress negative responses.

As these studies show, left-limb preference may serve as a predictor of susceptibility to the risk of poor welfare because it shows that the right hemisphere is controlling behavior and that hemisphere expresses negative emotions (1, 2). Limb preference is relatively easy to measure, and with adaptations to species and task requirements, it could be used to determine which individuals might be at a greater risk of suffering from poor welfare (49) or which animals have already been exposed to poor welfare conditions. In support of the latter, Barnard et al. (50) found that the paw preference of dogs shifts toward the left in the 1^st^ week after they have been housed in the stressful conditions of a dog shelter. Limb preference during the performance of specific tasks might also indicate those tasks that cause acute stress. For example, Siniscalchi et al. (51) found that, when being loaded onto a truck, horses that stepped onto the loading tray with their left forelimb (reflecting the use of the right hemisphere) displayed higher levels of anxious behavior than those that stepped onto the tray with their right forelimb.

### 2.2. Strength of lateralization and behavior

The strength of laterality is also associated with differences in behavior between individuals, and it serves as a marker of behavior important for welfare. One study compared the behavior of domestic chicks lateralized for visual processing with chicks lacking this lateralization. Since visual lateralization in chicks is generated by exposing the developing embryo *in ovo* to light (52–54), incubating eggs in darkness produces chicks without lateralization of visual pathways in the brain (4) and without lateralized visual behavior (52, 55). Chicks lacking visual lateralization have difficulty in performing a dual task, demanding the use of both hemispheres at the same time, the left hemisphere for food searching and the right hemisphere for detecting, and responding to a model predator (9). Compared with visually lateralized chicks, chicks hatched from eggs incubated in the dark and tested on the dual task are unable to find food scattered among pebbles and are less able to detect the predator moving overhead, although they have no difficulty performing either of these tasks when they are tested on them non-simultaneously. These findings showed that having a lateralized brain is advantageous in environments that require simultaneous attention to more than one type of stimulus, which is most often the case in the natural environment, as well as in most captive, living conditions (e.g., in agriculture). Moreover, chicks lacking visual lateralization make more distress calls once they do see the predator, showing that they are more disturbed by the presence of the predator (8). Their attention is also more easily distracted from the performance of a trained task (54). These results demonstrate the advantage of having a lateralized brain with functions separated to each hemisphere, thus reducing interference between hemispheres and thereby enhancing welfare.

Strength of limb preference can be used as a proxy for the degree of bias to use one hemisphere in preference to using both hemispheres. For example, dogs without a significant paw preference (with an ambilateral preference), measured on the Kong test (see above), react more strongly to the sounds of a thunderstorm than dogs do with left- or right-paw preferences (56). Ambilateral dogs are also found to be both more playful and more aggressive than dogs with significant paw preferences (57). Cats too with ambilateral paw preference have been found to be more aggressive and less affectionate than left- or right-pawed cats (58). However, another study found that ambilateral and right-pawed dogs had higher levels of stranger-directed aggression than left-pawed dogs (59). Further research on this topic is needed (see below), but, taken together, these findings indicate that separating brain functions into different hemispheres may reduce aggression.

Considering how the experience of stressful environments might affect paw preference and hemispheric dominance, Demirbas et al. (60) measured paw preferences in dogs housed under several different conditions and found that those that experienced stressful conditions were ambilateral, whereas dogs that had not experienced such chronic stress had significant paw preferences. Acute stress can also change the paw preference of dogs, as shown by a shift toward ambilaterality following a stressful open-field test (61). In other words, both acute stress and chronic stress affect paw preference, which, in turn, reflects the balance of hemispheric control. Paw preference in these examples can be used to make welfare decisions about living/housing conditions. As another example of ambilateral preference signaling poor conditions of welfare, in donkeys, reducing available living space was found to shift forelimb preference away from a right bias to no preference (62).

### 2.3. Applying knowledge of laterality to welfare

Measuring limb preference is a way to assess an animal's emotional state and, hence, how the animal will respond to stressful situations. A problem with using limb preference to assess welfare is deciding on the best method to measure it in different species. In the examples discussed above, limb preference in horses and donkeys has mostly been scored as the forelimb used to initiate movement or the forelimb advanced in front of the other forelimb while feeding. In marmosets, limb preference has been measured as the preferred hand used to hold food and take it to the mouth when feeding and, in dogs, as the preferred forelimb to hold down a Kong while licking food from it. Recently, there has been debate about the most reliable test for scoring limb preferences in dogs (63). For example, the first limb used by dogs when stepping off from a standing position has been compared with limb preference determined in the Kong test (57), with both measures showing similar, but not identical, differences in behavior between ambilateral dogs and dogs with significant limb preferences. As mentioned earlier, Barnard et al. (57) found that dogs scored as ambilateral on the Kong test were more playful and aggressive. The same study showed that dogs scored as ambilateral on the stepping test had higher scores on sociability and shy-boldness traits but not on aggressiveness. Deciding on the most reliable test of limb preference in dogs will be valuable if this measure is to become a standard indicator of the emotional state used to assess welfare in dogs.

Since species vary in their development of brain laterality and strength of functional laterality (64, 65), tests used to assess limb preferences relevant to welfare will need to be designed to accommodate species differences and differences in the conditions in which the animals are tested. It is clear that it will be necessary not only to separate the left-limb- from the right-limb-preferring individuals but also to determine which individuals are ambilateral. Concerning decisions on welfare, at present, the main body of evidence shows that left-limb-preferring and ambilateral individuals are at the most risk of poor welfare and/or are most likely to have experienced either acute or chronic stressful conditions.

Asymmetry of motor behavior other than limb preference may be used to indicate which hemisphere an animal is using in a particular context. One of these behaviors is side bias in tail wagging of dogs: A greater angle of tail wagging to the dog's left side reflects the use of the right hemisphere and this occurs when dogs see a dominant conspecific (negative response), whereas wagging the tail to a greater angle on the right side (the use of the left hemisphere) occurs when a dog sees its owner (positive response) (66). Dogs attend to and respond to tail wagging by conspecifics, as shown by increased anxious behavior when a dog sees another dog wagging its tail to its left side (67). Humans too could quite easily read these signals to assess the welfare of dogs.

Eyebrow movement might be another measure useful in assessing the emotional state of some breeds of dogs, as shown by Nagasawa et al. (68). These researchers found that, on being reunited with their owners, dogs displayed increased movement of the left eyebrow. Noting that the efferent nerves (trigeminal) to each side of the face cross the midline, movement of the left eyebrow is controlled by the output from the right hemisphere. It is not clear why being joined by their owner activated the dog's right hemisphere, although the finding is consistent with the specialization of the right hemisphere for social behavior. This measure of asymmetry deserves more experimental testing with potential application to welfare assessment.

This paper has, so far, focused on the functions of the right hemisphere since this hemisphere expresses negative emotions and behavior, potentially indicating poor welfare, but the converse can also be of value (i.e., the use of the left hemisphere to express positive response). A recent study has reported the preferential use of the left hemisphere by feral horses expressing positive, affiliative behavior (69). Consistent with this, d'Ingeo et al. (70) have found that horses react by using their left hemisphere when they hear human voices that have been associated with positive experiences in the past (determined by electroencephalogram measurement and ear movements), whereas the right hemisphere is used to react to voices associated with negative past experiences. Goats also use the left hemisphere to respond to familiar, non-threatening sounds (71). In fact, piglets forced to view a positively conditioned stimulus with their left eye (right hemisphere) showed reduced positive assessment, and the same side bias has been reported in horses (72, 73). These results are consistent with other studies that reported that the right hemisphere has a more negative assessment of stimuli and conditions (74). Taken together, these studies on several different species support the pattern of positive assessment by the left hemisphere and negative assessment by the right hemisphere.

The importance of the right hemisphere's involvement in social behavior also needs consideration in welfare. As studied in sheep, the right hemisphere is used to process faces (as mentioned above). Seeing images of faces of other sheep has a calming effect, and this is accompanied by a lowering of cortisol levels and increased activity in the amygdala of the right hemisphere, a region of the brain important in mediating fear responses, and in regions of the right hemisphere important in controlling emotions (16). The researchers suggest that images of faces could be used to reduce stress generated by social isolation (16). One could extend this suggestion to predict that having familiar conspecifics on the sheep's left side would be more calming than having them on the right side. Catering for this asymmetry could be useful in reducing the stress of herding and shearing and of moving sheep in runways or in small spaces, as encountered when they are loaded onto trucks.

## 3. Strategies to enhance control by the left hemisphere

Lateralization of the brain and behavior is not solely determined by genes, and it can be modified by both long-term and short-term influences. This flexibility means that laterally biased behavior can indicate conditions of acute and chronic stress. In fact, somewhat prolonged stress caused by deprivation of food has been shown to elevate the activity of the right hemisphere, as Pereira et al. (75) found in marmosets by measuring tympanic membrane temperature. Such flexibility in lateralization could be utilized to develop strategies to alter the strength and direction of laterality, thereby altering the reactions of animals to specific conditions and events (76). One approach to improve the welfare of chickens is to expose the eggs to light during incubation. As discussed above, light during incubation generates visual lateralization and alters the responses of the hatched chicks in conditions demanding attention to multiple stimuli (9). Archer and Mench (77) have examined the welfare aspects of this finding and found that daily exposure to 12 h of light during incubation leads to a long-lasting reduction of fear in chickens via an effect on their lateralization.

## 4. Conclusion

Limb preference, as an indicator of hemisphere bias or laterality expressed in specific behavioral contexts, has been discussed. It is increasingly evident that left-limb preference (right hemisphere in control) in a range of species is associated with heightened negative responding and, hence, poor welfare. Left-limb preference indicates those animals that are more likely to suffer stress, or it reflects past or ongoing exposure to poor welfare conditions. The strength of limb preference is also an indicator of coping ability. Weakly lateralized or non-lateralized individuals are less able to attend to more than one stimulus than are strongly lateralized individuals. They show higher levels of distress and impaired task performance in contexts requiring attention to multiple inputs.

Stress during early life may cause a long-lasting bias for the right hemisphere to control behavior and, consequently, negative cognitive bias (78, 79), which is expressed as anxiety and is associated with changes in neurotransmission in the right hemisphere (80). Further investigation is needed to find ways of reversing right hemispheric dominance and to permit the left hemisphere to take a larger, and preferably dominant, role in controlling behavior. Left hemisphere control is especially important for good welfare since the left hemisphere can suppress the activity of the right hemisphere (78) (see Figure 1). Enrichment and play have been suggested as likely routes to establish left-hemisphere control (78, 81, 82), and future research could investigate this. Further study of laterality in domesticated and companion animals offers the possibility of new insights into improving animal welfare.

## Author contributions

The author confirms being the sole contributor of this work and has approved it for publication.
